# New record and dietary ecology of a poorly-known treefrog, *Zhangixaluspachyproctus* Yu, Hui, Hou, Wu, Rao & Yang, 2019 (Anura, Rhacophoridae) in Vietnam

**DOI:** 10.3897/BDJ.12.e137528

**Published:** 2024-12-05

**Authors:** Anh Van Pham, Gam Thi Trinh, Linh Hai Thi Dang, Duyen Thi Duong, Truong Quang Nguyen, Minh Duc Le, Thomas Ziegler

**Affiliations:** 1 Faculty of Environmental Sciences, University of Science, Vietnam National University, Ha Noi, 334 Nguyen Trai Road, Hanoi, Vietnam Faculty of Environmental Sciences, University of Science, Vietnam National University, Ha Noi, 334 Nguyen Trai Road Hanoi Vietnam; 2 Tay Bay University, Quyet Tam Ward, Son La, Vietnam Tay Bay University, Quyet Tam Ward Son La Vietnam; 3 Institute of Ecology and Biological Resources, Vietnam Academy of Science and Technology, 18 Hoang Quoc Viet Road, Hanoi, Vietnam Institute of Ecology and Biological Resources, Vietnam Academy of Science and Technology, 18 Hoang Quoc Viet Road Hanoi Vietnam; 4 Graduate University of Science and Technology, Vietnam Academy of Science and Technology, 18 Hoang Quoc Viet Road, Hanoi, Vietnam Graduate University of Science and Technology, Vietnam Academy of Science and Technology, 18 Hoang Quoc Viet Road Hanoi Vietnam; 5 Central Institute for Natural Resources and Environmental Studies, Vietnam National University, Hanoi, 19 Le Thanh Tong, Hanoi, Vietnam, Hanoi, Vietnam Central Institute for Natural Resources and Environmental Studies, Vietnam National University, Hanoi, 19 Le Thanh Tong, Hanoi, Vietnam Hanoi Vietnam; 6 Department of Herpetology, American Museum of Natural History, Central Park West at 79th Street, New York, USA, Virgin Islands (USA) Department of Herpetology, American Museum of Natural History, Central Park West at 79th Street New York, USA Virgin Islands (USA); 7 Institute of Zoology, University of Cologne, Zülpicher Street 47b, 50674, Cologne, Germany Institute of Zoology, University of Cologne, Zülpicher Street 47b, 50674 Cologne Germany; 8 AG Zoologischer Garten Köln, Riehler Straße 173, 50735 Köln, Cologne, Germany AG Zoologischer Garten Köln, Riehler Straße 173, 50735 Köln Cologne Germany

**Keywords:** Lang Son Province, invertebrates, prey items, Son La Province, stomach contents

## Abstract

**Background:**

The White-lipped Treefrog *Zhangixaluspachyproctus* Yu, Hui, Hou, Wu, Rao & Yang, 2019 was previously confused with *Zhangixalussmaragdinus* (Blyth, 1852). Records of *Zhangixalussmaragdinus* from Yunnan (China), Vietnam and Thailand were recognised as a misidentification and this taxon was subsequently described as a new species, based on morphological and molecular evidence. This species is currently known from southern China, Thailand, Laos and Vietnam. However, knowledge about its natural history and feeding ecology is virtually lacking.

**New information:**

We herein report a newly-discovered population of *Zhangixaluspachyproctus* from Lang Son Province, northern Vietnam. In addition, we provide novel data about the diet of *Z.pachyproctus*, based on stomach content analyses of 38 individuals (37 males and one female). A total of 26 prey categories with 681 items were found in the stomachs of *Z.pachyproctus*. The dominant prey items of the species were Orthoptera (Acrididae, Tettigoniidae), Coleoptera (Languriida, Leiodidae, larvae, other Coleoptera), Mantodea (Mantidae) and Blattodea (Blaberidae, Blattidae, other Blattodea). Coleoptera (Coccinellidae, Cupedidae, Elateridae, Languriidae Leiodidae, Tenebrionidae, larvae and other Coleoptera) was the category with the highest frequency of prey items, found in 24 stomachs. The most important (IRI) groups amongst the prey of *Z.pachyproctus* were Coleoptera (24.52%) and followed by Orthoptera (24.43%), Blattodea (9.12%), Mantidae (8.14%) and Hemiptera (4.05%).

## Introduction

The genus *Zhangixalus* Li, Jiang, Ren & Jiang, 2019 currently contains 44 recognised species, distributed from India, Nepal, Bhutan, China (including Taiwan), Myanmar, Thailand, Laos, Vietnam, Japan, Indonesia, Brunei and Malaysia (Frost 2024). *Zhangixalus* is a poorly-known group of treefrogs with seven new species having been discovered over the last five years, viz. *Zhangixaluspachyproctus* Yu, Hui, Hou, Wu, Rao & Yang, 2019; *Z.franki* Ninh, Nguyen, Orlov, Nguyen & Ziegler, 2020; *Z.jodiae* Nguyen, Ninh, Orlov, Nguyen & Ziegler, 2020; *Z.melanoleucus* Brakels, Nguyen, Pawangkhanant, Idiiatullina, Lorphengsy, Suwannapoom & Poyarkov, 2023; *Z.faritsalhadii* Gonggoli, Munir, Kaprawi, Kirschey & Hamidy, 2024; *Z.thaoae* Nguyen, Nguyen, Ninh, Le, Bui, Orlov, Hoang & Ziegler, 2024; and *Z.yunnanensis* Pan, Hou, Yu & Liu, 2024 (Yu et al. 2019, Nguyen et al. 2020, Ninh et al. 2020, Peter et al. 2023, Pan et al. 2024, Nguyen et al. 2024). In Vietnam, Frost (2024) listed 12 species of *Zhangixalus*, namely *Z.dennysi*, *Z.dorsoviridis*, Z. *duboisi*, *Z.dugritei*, *Z.feae*, *Z.franki*, *Z.hungfuensis*, *Z.jodiae*, *Z.nigropunctatus*, *Z.pachyproctus*, *Z.puerensis* and *Z.thaoae*. Of these, *Zhangixaluspachyproctus* is known to occur in Thailand, China, Laos and Vietnam (Frost 2024). In Vietnam, *Zhangixaluspachyproctus* has been recorded from Son La, Bac Giang, Quang Ninh, Tuyen Quang, Cao Bang and Nghe An Provinces (Pham et al. 2017, Frost 2024). However, dietary ecology of *Z.pachyproctus* is unknown.

Studying the diet of frogs helps us understand an important aspect of their natural history and that is essential for successful conservation and management programmes. Previous studies have shown that frogs were considered opportunistic predators when their diet had a relationship with prey availability in the environment (Duellman and Trueb 1994), of which, insects are the most frequent prey category of frogs, with the richest species diversity (Werner et al. 1995, Barrasso et al. 2009, Caldart et al. 2012, Brito et al. 2013, Pham and Nguyen 2018, Pham et al. 2024). On the other hand, the number of studies on the diet of tree frogs is still limited, a few exceptions being Pham et al. (2022), who investigated the diet of *Zhangixalusfeae* (Boulenger, 1893) (Amphibia, Anura) in Son La Province; Pham et al. (2023) who studied the feeding ecology of the big-headed treefrog, *Polypedatesmegacephalus* from north-western Vietnam; and Trinh et al. (2023) who examined segregation in diet composition of two syntopic tree frog species, *Hylasimplex* and *Polypedatesmegacephalus* in Ben En National Park, Vietnam.

In this study, we investigated the diet composition of *Zhangixaluspachyproctus* from Xuan Nha and Huu Lien Natural Reserves, Vietnam. In addition, we report the first record of *Z.pachyproctus* from Lang Son Province, Vietnam.

## Materials and methods

**Sampling**

Field surveys were conducted at two sites: (1) Xuan Nha Nature Reserve (NR), Son La Province, north-western Vietnam (21°20'020"N 103°34'302"E, elevation: 1260 m a.s.l.) from 1 to 5 May 2021 and (2) Huu Lien NR, Lang Son Province, north-eastern Vietnam (20°37'242"N, 104°41'369"E, elevation: 610 m a.s.l.) from 3 to 14 May 2023.

Frogs were captured by hand at ponds (approximately 30.0–50.0 m in length and 20.0–40.0 m in width) between 20:30 h and 24:00 h following the guidelines approved by the American Society of Ichthyologists and Herpetologists for animal care (Beaupre et al. 2004). Frogs were found on the ground or on tree branches at the edges of ponds in the secondary forest. The surrounding habitat was second forest with medium and small wood and shrubs. Air temperature was 25-32°C and relative humidity was 70-85%. The stomach-flushing technique was used to obtain stomach contents without sacrificing frogs (Griffiths 1986, Leclerc and Courtois 1993, Solé et al. 2005). Prey items were preserved in 70% ethanol. Frogs were subsequently released at the collecting site after measurements of snout-vent length (SVL) and mouth width (MW) with a digital caliper to the nearest 0.01 mm and measured weight (BM) using electronic scales to the nearest 0.1 g.

**Species identification**

For taxonomic identification, two individuals were collected for voucher specimens. Field surveys were permitted by the Directorates of Xuan Nha and Huu Lien NRs (permit No. 126/KBTTNXN issued on 27 April 2021 and permit No. 126/KBTTNHL issued on 3 May 2023). After having been photographed in life, animals were anaesthetised and euthanised in a closed vessel with a piece of cotton wool containing ethyl acetate (Simmons 2002), fixed in 85% ethanol and subsequently stored in 70% ethanol. Measurements were taken with a digital calliper to the nearest 0.1 mm. Abbreviations are as follows: a.s.l.: above sea level; terminology of morphological characters followed Yu et al. (2019): SVL (snout-vent length): from tip of snout to vent; HL (head length): from posterior corner of mandible to tip of snout; MW (mouth width): at the angle of jaws; IND (internarial distance); DNE: distance from anterior corner of eye to posterior edge of nostril; ED (eye length); TD (maximum tympanum diameter); DTE: distance between anterior margin of tympanum and posterior corner of eye; SL: distance from anterior corner of eye to tip of snout; IOD (minimum distance between upper eyelids); UEW (maximum width of upper eyelid); FHL (hand length): from elbow to tip of third finger; THL (thigh length): from vent to knee; TL (tibia length); FL (foot length): from proximal end of inner metatarsal tubercle to tip of fourth toe; TFL (combined length of foot and tarsus): from base of inner metatarsal tubercle to tip of fourth toe.

**Stomach content analysis**

Prey items were identified under a microscope (Olympus SZ 700), based on identification keys (i.e. Naumann et al. (1991), Johnson and Triplehorn (2005), Brusca et al. (2016), Thai (2022)). The maximum length (L) and width (W) of each prey item were measured to the nearest 0.1 mm using either a caliper or a calibrated ocular micrometer fitted to a microscope. Body parts of the same individual were assembled before taking measurements, otherwise prey with incomplete bodies were measured separately. The volume (V) of prey item was calculated using the formula for a prolate spheroid (π = 3.14; Pham et al. (2022)): V = (4π/3)×(L/2)×(W/2) (mm³). To evaluate the relative importance of each prey category, we calculated the following three indices: %F, the frequency of occurrence (the percentage of stomachs containing a specific prey category amongst stomachs containing prey categories); %N, the relative number (the percentage of a specific prey category amongst the number of the bulk of prey categories); and %V, the relative volume (the percentage of the volume of a specific prey category amongst the volume of the bulk of prey categories (Nakamura and Tominaga 2021).

The index of relative importance (IRI) was used to determine the importance of each food category. This index provides a more informed estimation of prey item consumption than any of the three components alone by using the following formula (Caldart et al. 2012): IRI = (%F + %N + %V)/3, where F is the frequency of prey occurrence in stomachs and N is the total number of prey items concerning all prey items.

We used the reciprocal Simpson’s heterogeneity index, 1-D, to calculate dietary heterogeneity: D = ∑[n_i_(n_i_ – 1)]/[N(N – 1)], where n_i_ is the number of prey items in the i^th^ taxon category and N is the total number of prey items (Pham et al. 2024).

To estimate prey evenness, we used Shannon’s index of evenness. Evenness is calculated from the equation: J’ = H’/H_max_ = H’/ln S. The maximum diversity (H_max_) that could occur is that which would be found in a situation in which all taxa had equal abundance (H’ = H_max_ = ln S), S is the total number of prey taxa and H’ is the Shannon-Weiner index of taxon diversity. The value of H’ is calculated from the equation: H′ = –∑(Pi×lnPi),

where Pi is the ratio of food items in the taxon to the total number of food items in the sample (Magurran 2004, Muñoz-Pedreros and Merino 2014).

We used linear regression to examine the relationship between mouth width (MW), snout-vent length (SVL), measured weight (BM) and prey size, as well as prey volume (Nakamura and Tominaga 2021).

Statistic analyses were performed using software package SPSS 20.0 (SPSS Inc., Chicago, Illinois, USA) and with the significance level set to *P* < 0.05 for all analyses. Data were presented as mean ± standard deviation (SD) unless otherwise noted. We used Kendall’s_tau b statistics to examine the number of prey items, prey volume from frogs of different sexes. We used one-way analysis of variance (ANOVA) to examine the size of prey items and the SVL, MW and BM. Symbols: r is the correlation coefficient; the F_1_-value is an analysis of variance (ANOVA) test between two groups; the p-value represents the probability of obtaining a different result.

## Taxon treatments

### Zhangixalus pachyproctus

Yu, Hui, Hou, Wu, Rao & Yang, 2019

7540F22D-3F91-54C2-8596-18A959158DE8

#### Materials

- **Type status:** Other material. **Occurrence:** catalogNumber: LS.2023.36; individualCount: 1; sex: male; occurrenceID: 43C1E6EB-F58E-5F5E-B999-9636D9DC169C; **Taxon:** scientificNameID: *Zhangixaluspachyproctus*; scientificName: *Zhangixaluspachyproctus*; class: Amphibia; order: Anura; family: Rhacophoridae; genus: Zhangixalus; specificEpithet: *pachyproctus*; scientificNameAuthorship: Yu, Hui, Hou, Wu, Rao, and Yang, 2019; **Location:** country: Vietnam; countryCode: VN; stateProvince: Lang Son; county: Lang Son; municipality: Bac Son; locality: Near Tran Yen Commune; verbatimElevation: 610; verbatimLatitude: 20°20'020"N; verbatimLongitude: 103°34'302"E; verbatimCoordinateSystem: WGS84; **Event:** eventDate: May; eventTime: 2023; eventRemarks: collected by A.V. Pham; **Record Level:** language: en; collectionCode: Amphibia; basisOfRecord: PreservedSpecimen
- **Type status:** Other material. **Occurrence:** catalogNumber: LS.2023.45; individualCount: 1; sex: male; occurrenceID: 2B6B36F7-B099-54A1-A5E3-2F1A6FD4196E; **Taxon:** scientificNameID: *Zhangixaluspachyproctus*; scientificName: *Zhangixaluspachyproctus*; class: Amphibia; order: Anura; family: Rhacophoridae; genus: Zhangixalus; specificEpithet: *pachyproctus*; scientificNameAuthorship: Yu, Hui, Hou, Wu, Rao, and Yang, 2019; **Location:** country: Vietnam; countryCode: VN; stateProvince: Lang Son; county: Lang Son; municipality: Bac Son; locality: Near Tran Yen Commune; verbatimElevation: 610; verbatimLatitude: 20°20'020"N; verbatimLongitude: 103°34'302"E; verbatimCoordinateSystem: WGS84; **Event:** eventDate: May; eventTime: 2023; eventRemarks: collected by A.V. Pham; **Record Level:** language: en; collectionCode: Amphibia; basisOfRecord: PreservedSpecimen

#### Description

**Figure 1.**
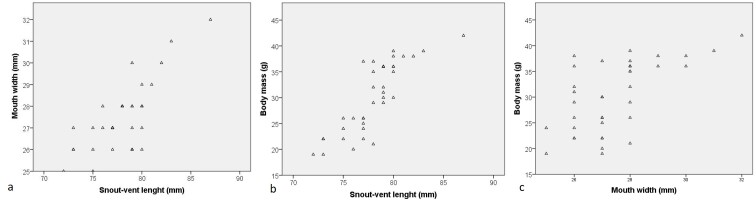
Dispersion diagrams from Pearson’s correlations between (a) snout-vent length and mouth width, (b) snout-vent length and body mass and (c) mouth width and body mass of *Zhangixaluspachyproctus*, Huu Lien Nature Reserve, Vietnam.

Morphological characters of specimens from Vietnam agreed well with the description of Pham et al. (2017) and Yu et al. (2019): SVL min-max: 71.9–83.4 mm; mean and SD: 77.81 ± 2.66 mm, n = 37), MW 24.9–31.2 mm (27.29 ± 1.26 mm, n = 37) and body mass (BM 18.6–39.0 g, 29.49 ± 6.76 g, n = 37) in males was smaller than that in the female SVL 87.5 mm; MW 31.9 mm; BM 42.5 g. There was a significant relationship between SVL, MW and BM (SVL and HW: *F*_1,41_ = 7.085, *P* < 0.001, *R^2^* = 0.733; SVL and BM: *F*_1,41_ = 13.408, *P* < 0.001, *R^2^* = 0.825; and HW and BM: *F*_1,41_ = 2.272, P = 0.040, *R^2^* = 0.613 (Fig. 1). Snout round; loreal region sloping, slightly concave; nostril oval, slightly protuberant, closer to snout tip than to eye (SNL 4.2–4.4 mm, DNE : 5.0–5.1 mm, n = 2, males); internarial distance narrower than interorbital distance and wider than upper eyelid width (IND 8.4–8.6 mm, IOD 9.2–9.5 mm, UEW 6.4–6.8 mm); tympanum distinct, round, more than half eye diameter (TD 4.8–5.2 mm, ED 7.3–7.7 mm) (Table 1); vomerine teeth present, in two series, touching inner front edges of choanae, separated from each other; tongue attached anteriorly, deeply notched posteriorly; internal single vocal sac present in males.

Forelimbs: FHL 38.3–38.5 mm; relative length of fingers I < II < IV < III; tips of all fingers expanded into discs with circum-marginal and transverse ventral grooves; nuptial pads present on first and second fingers; fingers webbed (Table 1).

Hind-limbs: Thigh shorter than tibia and foot (THL 35.9–36.4 mm, TL 36.9–37.1 mm, FL 36–36.6 mm); relative length of toes I < II < III < V < IV; tips of toes expanded into discs with circum-marginal and transverse ventral grooves; toes fully webbed; inner metatarsal tubercle distinct, oval; outer metatarsal tubercle absent; heels slightly overlapping when legs at right angle to body (Table 1).

Skin. Dorsal skin smooth; supratympanic fold distinct, curving from posterior edge of eye to insertion of arm; throat and chest smooth, flanks, belly and ventral surface of thighs granular; a narrow dermal fringe along outer edge of tarsus and fifth toe; vent protruding, forming an arc-shaped swelling.

**Figure 2.**
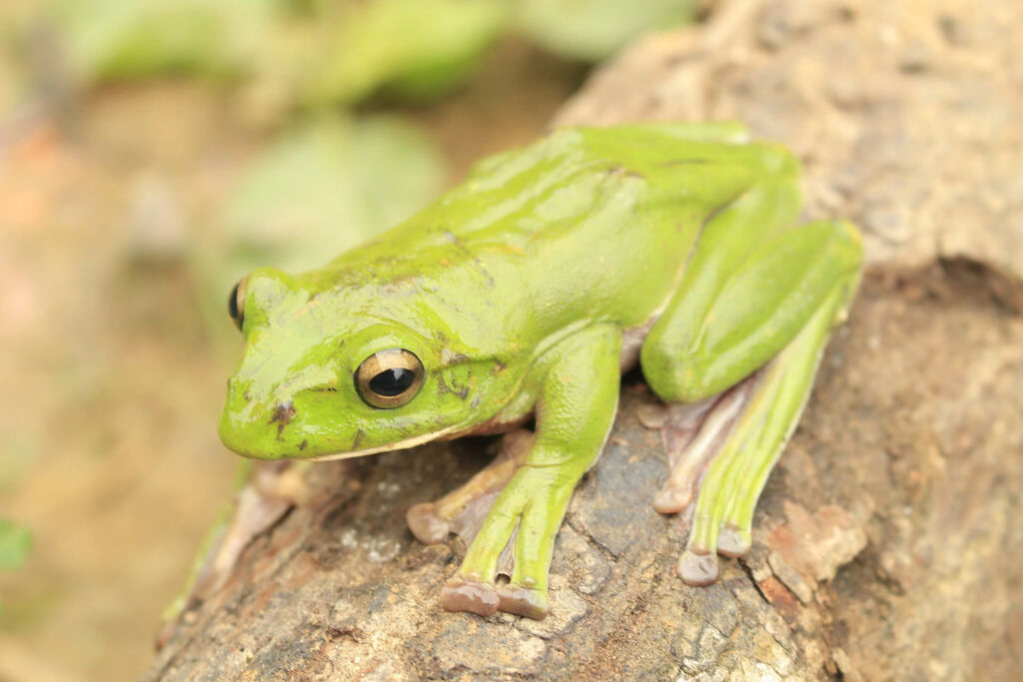
Adult male of *Zhangixaluspachyproctus*, Huu Lien Nature Reserve, Vietnam.

**Figure 3.**
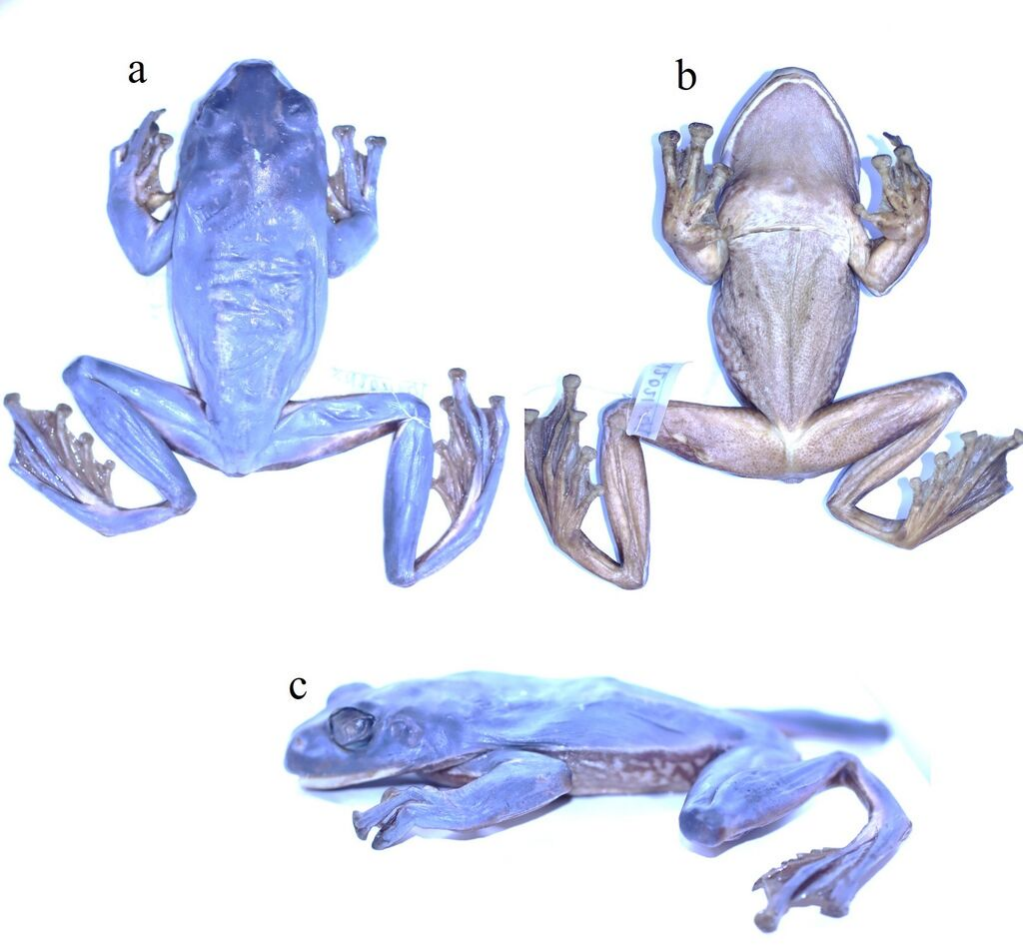
*Zhangixaluspachyproctus* in preservative, adult male (LS.2023.36): **a** dorsal view; **b** ventral view; **c** lateral view.

**Figure 4.**
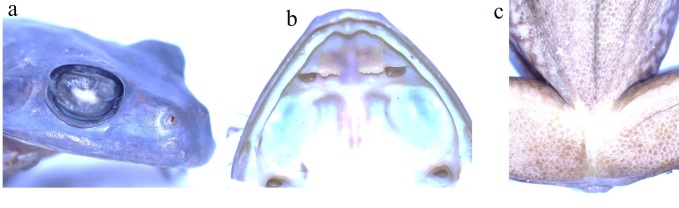
*Zhangixaluspachyproctus* in preservative, adult male (LS.2023.36): **a** lateral view of head; **b** vomerine teeth arrangement; **c** ventral view of cloacal region.

Colouration in life. Dorsal surface green with some yellow dots; supratympanic fold and flanks light green; with a light stripe from the margin of the lower jaw to the groin; ventral surface of body and limbs grey white; lower part of flanks, abdomen and ventral surfaces of hind-limbs scattered with clouded light brown spots (Fig. 2). Colouration in preservative, see (Fig. 3) and (Fig. 4).

Ecology notes. This species was found on the ground or on tree branches, about 0.5–2.5 m above the forest floor, near ponds. Surrounding habitat was mixed evergreen forest of small hardwood, bamboo and shrub at elevations between 500 and 1110 m.

#### Distribution

In Vietnam, this species was recorded from Son La, Tuyen Quang, Bac Giang, Cao Bang, Quang Ninh and Nghe An Provinces (Pham et al. 2017, Frost 2024). Elsewhere, this species is known from China, Thailand and Laos (Frost 2024). This is a new record for Lang Son Province.

#### Diet

A total of 38 adult individuals (37 males and 1 female) of *Z.pachyproctus* was collected from Son La and Lang Son Provinces. We identified 681 prey items for sampled *Z.pachyproctus*, including 680 prey items in males and one prey item in the female.

The number of prey items per individual was 1–64 items (average 17.92 ± 16.58 items).

Mean prey item length was 3.41 ± 4.11 mm (ranging from 0.90 to 45.00 mm) and mean prey item width was 1.18 ± 1.06 mm (ranging from 0.60 to 12.00 mm) in both sexes. The average volume per individual was 245.38 ± 424.71 mm^3^ (ranging from 0.26 to 1544.10 mm^3^).

**Figure 5.**
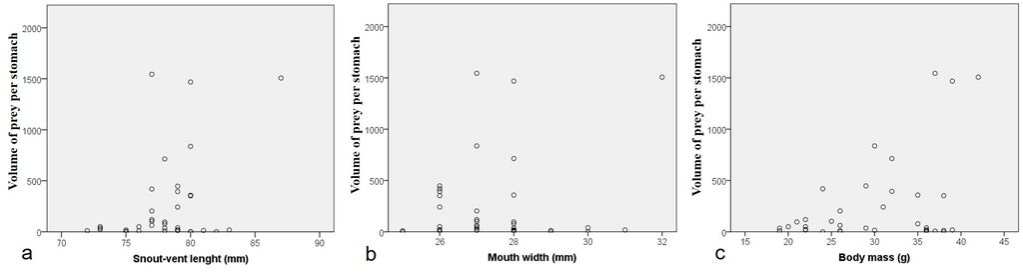
Relationships between prey volume per stomach (a) snout-vent length, (b) snout-vent length and (c) body mass of *Zhangixaluspachyproctus*, Huu Lien Nature Reserve, Vietnam.

There was no positive correlation between the frog’s SVL, MW, BM and the prey volume per individual (SVL, Kendall’s tau b: tau = 0.107, *P* = 0.366; MW: tau = - 0.089, *P* = 0.467; BM: tau = 0.043, *P* = 0.714) (Fig. 5).

We identified 12 prey categories in the stomachs of *Z.pachyproctus*. Insects were the main food component of *Z.pachyproctus*, with nine categories and other invertebrates (Oligochaeta, Scolopendromorpha and Gastropoda) (Table 2).

**Figure 6.**
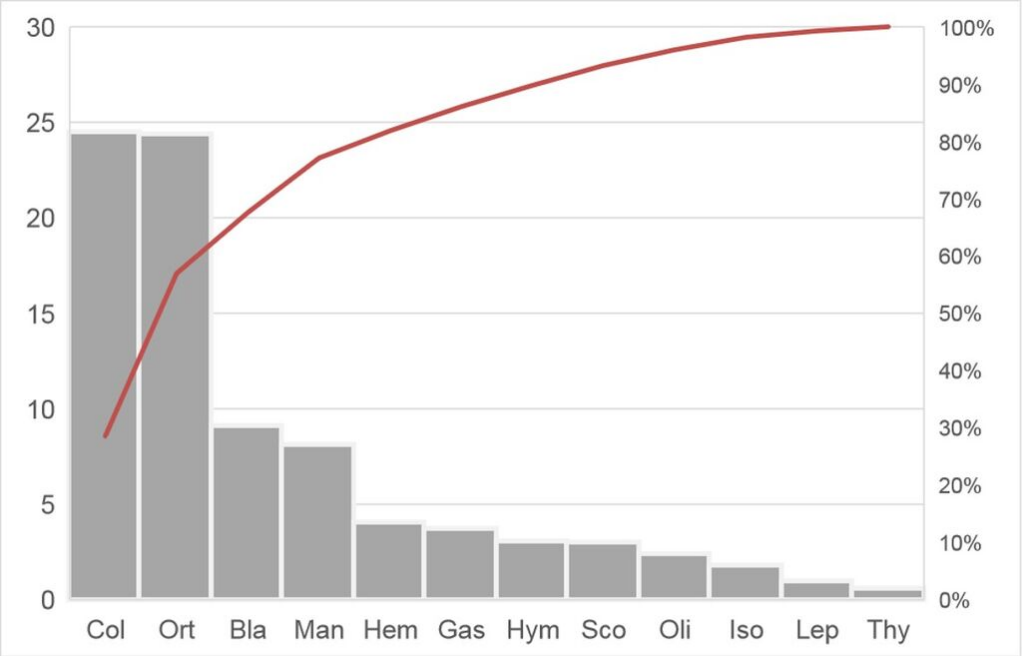
Importance indices (IRI) for prey categories consumed by *Zhangixaluspachyproctus* in Vietnam. Coleoptera (Col), Orthoptera (Ort), Blattodea (Bla), Mantodea (Man), Hemiptera (Hem), Gastropoda (Gas), Hymenoptera (Hym), Scolopendromorpha (Sco), Oligochaeta (Oli), Isoptera (Iso), Lepidoptera (Lep), Thysanura (Thy).

The highest number of prey items found was Orthoptera (41.56%), followed by Coleoptera (25.99%), Mantodea (9.1%), Blattodea (8.37%), Hemiptera (7.05%) and Hymenoptera (4.99%). While the most frequently foraged prey group was Coleoptera (40.0%), followed by Orthoptera (20.0%), Blattodea (10.0%) and Hemiptera (5.0%). In terms of IRI, Coleoptera (24.52%), followed by Orthoptera (24.43%), Blattodea (9.12%), Mantidae (8.14%) and Hemiptera (4.05%) were found (Fig. 6). The total dietary breadth of *Z.pachyproctus* from Vietnam was 0.89 (Simpson’s index of diversity) and Shannon’s evenness was 0.77.

## Discussion

*Zhangixaluspachyproctus* is the sister taxon of *Z.smaragdinus* (Frost 2024). Yu et al. (2019) excluded records of *Z.smaragdinus* from Yunnan, Vietnam and Thailand and subsequently described the taxon from these localities as the new species *Z.pachyproctus*, based on morphological and molecular evidence. Specifically, *Z.pachyproctus* can be clearly distinguished from *Z.smaragdinus* by having the protruding posterior end of the body in males that forms an arc-shaped swelling (versus vent not protruding in *Z.smaragdinus*); interspace between vomerine ridges narrower than that in *Z.smaragdinus*; large grey reticular mottles below the white stripe on flank (versus fine in *Z.smaragdinus*) and more sloped snout in profile (Yu et al. 2019) (Figs 3, 4).

Most tropical-subtropical frogs have often been reported feeding on spiders, cockroaches, beetles, grasshoppers and ants (e.g. Biavati et al. (2004), Quiroga et al. (2009), Brito et al. (2013), Pham and Nguyen (2018), Pham et al. (2024)). The diet of *Zhangixaluspachyproctus* was primarily composed of invertebrates, with cockroaches, beetles, crickets, grasshoppers, ants and termites, which is nearly similar to the diet of many frogs in Vietnam (Pham and Nguyen 2018, Pham et al. 2023, Pham et al. 2024). In the population of *Polypedatesmegacephalus* from north-western Vietnam studied by Pham et al. (2023), the diet was dominated by arthropods (Orthoptera, Isopoda, Araneae, Coleoptera, Opiliones and Hymenoptera) and the most important prey of *Z.pachyproctus* (Orthoptera, Coleoptera, Blattidae and Mantodea) were absent or had little importance for *Z.pachyproctus*. Despite differences in diet between *Z.pachyproctus* and *P.megacephalus*, Orthoptera and Coleoptera represented the most important prey categories for the two species. On the other hand, Pham et al. (2022) reported the dietary composition of *Zhangixalusfeae* (Boulenger 1893), another representative of the genus *Zhangixalus* from northern Vietnam. Both *Z.pachyproctus* and *Z.feae* inhabit similar environment conditions, viz. on the ground or tree branches near ponds or streams in evergreen forest with hardwood, bamboo and shrub (Pham et al. 2017, Pham et al. 2022). The prey categories of *Z.pachyproctus* were more diverse than those in *Z.feae* (12 vs. 10) (Pham et al. 2022). Interestingly, the prey categories of *Z.pachyproctus* are similar to those of *Z.feae*, with Blattodea, Coleoptera, Hemiptera, Lepidoptera, Orthoptera and Gastropoda (Pham et al. 2022). Nonetheless, Oligochaeta, Scolopendromorpha, Hymenoptera, Isoptera, Mantodea and Thysanura were found exclusively in the diet of *Z.pachyproctus*, whereas Araneae, Dermaptera, Phasmatodea and Aves occurred only in the diet of *Z.feae* (Pham et al. 2022). The differences in the diet between *Z.pachyproctus* and *Z.feae* may reflect differences in foraging activity and microhabitat use. In this study, we only collected food from one female and only one prey category (Gastropoda) was found in the female's stomach. Further comprehensive studies on the diet of both sexes of *Z.pachyproctus* are needed in the future.

## Supplementary Material

XML Treatment for Zhangixalus pachyproctus

## Figures and Tables

**Table 1.** Measurements (in mm) of *Zhangixaluspachyproctus* collected from Vietnam in comparison with the type series from China.

	LS2023.36	LS2023.45	Yu et al. (2019)
Sex	Male	Male	Males (Min-Max)
SVL	77.6	76.7	73.4–78.2
HL	23.5	23.1	21.9–24.1
HW	28.6	27.6	26.6–29
SL	12.6	12	11.7–12.8
IND	8.6	8.4	7.9–8.9
1OD	9.5	9.2	8.1–9.7
UEW	6.8	6.4	6.2–6.8
ED	7.7	7.3	6.9–8.8
TD	5	4.8	4.6–5.1
DNE	5.1	5	4.9–5.4
DTE	2.3	2.2	2.2–2.5
FHL	38.5	38.3	36.3–39.8
THL	36.4	35.9	33–37.3
TL	37.1	36.9	34.3–38.3
FL	36.6	36	34.2–37.8
TFL	54	52.9	49.1–54.9
TL/SVL	0.48	0.48	0.46–0.51
HL/SVL	0.30	0.30	0.29–0.32
HL/HW	0.82	0.84	0.78–0.87
SL/ED	1.64	1.64	1.39–1.7
ED/UEW	1.13	1.14	1.11–1.31

**Table 2.** Prey categories consumed by *Zhangixaluspachyproctus* in Vietnam (n = 38), (F) total frequency, (%F) relative frequency, (N) total abundance, (%N) relative abundance, (V) total volume (mm³), (%V) relative volume; (IRI) importance index.

	%F	%N	%V	IRI
Oligochaeta	3.33	0.29	5.45	3.02
Gastropoda	1.67	0.15	9.31	3.71
Scolopendromorpha	6.67	0.59	0.02	2.42
**Blattodea**	**10.00**	**8.37**	**8.99**	**9.12**
Blaberidae	3.33	3.38	0.37	2.36
Blattidae	1.67	3.23	0.12	1.67
other Blattodea	5.00	1.76	8.50	5.09
**Coleoptera**	**40.00**	**25.99**	**7.56**	**24.52**
Coccinellidae	15.00	11.01	3.25	9.75
Cupedidae	1.67	0.15	1.70	1.17
Elateridae	1.67	1.76	0.06	1.16
Languriidae	3.33	0.29	0.01	1.21
Leiodidae	1.67	0.15	0.00	0.61
Tenebrionidae	1.67	0.15	0.58	0.80
Larvae	5.00	0.88	1.22	2.37
other Coleoptera	10.00	11.60	0.74	7.45
**Hemiptera**	**5.00**	**7.05**	**0.09**	**4.05**
Pentatomidae	1.67	6.61	0.04	2.77
other Hemiptera	3.33	0.44	0.05	1.28
**Hymenoptera**	**3.33**	**4.99**	**0.88**	**3.07**
Formicidae	1.67	3.82	0.84	2.11
Scoliidae	1.67	1.17	0.04	0.96
**Lepidoptera**	**1.67**	**0.15**	**1.16**	**0.99**
Geometridae	1.67	0.15	1.16	0.99
**Mantodea**	**3.33**	**9.10**	**11.97**	**8.14**
Mantidae	3.33	9.10	11.97	8.14
**Orthoptera**	**20.00**	**41.56**	**11.75**	**24.43**
Acrididae	8.33	21.73	5.03	11.70
Gryllidae	3.33	2.20	5.31	3.62
Tettigoniidae	6.67	11.75	1.19	6.53
other Orthoptera	1.67	5.87	0.22	2.59
Isoptera	3.33	1.62	0.42	1.79
Thysanura	1.67	0.15	0.00	0.61
